# An Entrance Examination for Probationers

**Published:** 1920-07-31

**Authors:** 


### THE MATRONS' AND SISTERS' DEPARTMENT.

### AN ENTRANCE EXAMINATION FOR PROBATIONERS.

In the present dearth of candidates, to suggest the heroic method of making it more difficult instead of more easy to become a probationer causes some surprise. Yet that in effect was what Dr. Lane Claypon ventured to do at the College Conference in June. Many present believed it to be a counsel of wisdom, but few could see how it can be carried out. A glimmer of light begins to dawn however.

In every other profession worthy of the name a definite standard of education exists for entrants. No girl is admitted to study for a medical degree, for instance, unless she has matriculated. Would-be dispensers must pass a preliminary examination in English, Latin, a foreign language, etc., before commencing their apprenticeship, and so on. But when the headmistresses inquire, as they do sometimes, what standard of education is necessary for a nurse the only answer which can be made is "None."

Undoubtedly it would be a great gain if matrons and nurses in conclave could determine what general standard of education all probationers ought to have attained before the business of teaching them to become nurses begins. At least half the trials of sister-tutors arise from the necessity of supplementing the ordinary attainments of their pupils in subjects unconnected with nursing. What matron has not groaned in spirit over the bad writing, faulty spelling, grammatical solecisms, and lack of coherent expression displayed among her probationers? The need for levelling up is conspicuous and insistent. And yet, like many reforms needed in the nursing profession, to impose a standard for probationers can only be achieved very gradually and spread from the top downwards. By far the greater number of institutions are in such perplexity for workers that they are ready to give a cheerful welcome to all sane, healthy candidates offering good recommendations and will turn a blind eye to their deficiencies in grammar and spelling.

It is true that there are one or two hospitals which impose an entrance examination of varying rigour on applicants. They reap their reward in securing for the most part women well prepared to benefit by the theoretical as well as the practical courses of instruction provided. But there are several drawbacks to this system. In the first place the candidate does not know which institutions require her to pass an entrance examination until she applies for information. The hospitals in question are compelled to examine their own candidates, an unsatisfactory and costly course for a hospital to pursue, and thus time and money are wasted, while rejected candidates are discouraged. Some central authority is needed to examine and sift the candidates and where can we look but to the College of Nursing to undertake this duty?

Suppose there were a group of training-schools in London and the provinces able to lay down as a condition for entrants the production of a certificate, the Junior Cambridge or third class of the College of Preceptors, not to begin too high, would not this be a good start? It would not suffice, however, for there are many young women ready for employment who could rise to this standard, but have never had the chance of passing this or any other public examination. We come back to the nation we have before touched on of a department of the College of Nursing organised as a kind of clearing-house for candidates. Could not the College hold quarterly examinations for women desirous of becoming nurses and place the examinees in, say, three classes according to their attainments? We are quite sure that places could be found for all who presented themselves except such as displayed entire inaptitude, and even these might be secured for the domestic side. By these means women who have learned to use their brains could be drafted to institutions which would afford them the opportunity of doing so. And those who without special talent could yet hold their own in the examination room would be welcomed everywhere. The task of getting the right kind of probationer to fit into the school would be enormously facilitated.

In some such way the desired standard for entrants could be gradually established. There is not likely to be any finality about it. It seems probable that the whole educational standard of the country will be raised within the next ten years. And that for nurse candidates will rise with it. But once let it be known that education does matter to a nurse, that the best training-schools cannot be entered unless a definite standard has been attained, and the whole nursing outlook will undergo a transformation. When we are asked what degree of education a woman ought to have before she begins her training, at least let us have an answer to give.

